# Turnover Rates and Numbers of Exchangeable Hydrogens in Deuterated Water Labeled Samples

**DOI:** 10.3390/ijms26136398

**Published:** 2025-07-03

**Authors:** Henock M. Deberneh, Ali Bagherinia, Rovshan G. Sadygov

**Affiliations:** Department of Biochemistry and Molecular Biology, The University of Texas Medical Branch, 301 University of Blvd, Galveston, TX 77555, USA

**Keywords:** protein turnover, deuterated water labeling, the number of exchangeable hydrogens

## Abstract

Metabolic labeling with deuterated water is used in combination with liquid-chromatography coupled with mass spectrometry to study the turnover rates of individual proteins in vivo. This technique and bioinformatics tools for data analysis quantify the turnover rates of thousands of proteins. Turnover rates change during organismal growth and respond to alterations in the environment and diet. The accurate and statistically significant determination of the turnover rate changes of a protein depend on the variations in the turnover rates of the peptides of the protein. One of the systematic factors contributing to this variability is the dependence of the turnover rates on the number of exchangeable hydrogens of the peptides. This variability (by reducing the statistical power) reduces biological interpretability. Here, we propose a computational approach to eliminate the dependence of the turnover rates on the number of exchangeable hydrogens. This approach enhances the accuracy of turnover rate estimation and may help to support more accurate assessments of biological dynamics and disease mechanisms.

## 1. Introduction

Proteins are continuously synthesized and degraded. The process termed protein turnover removes non-functioning, old, damaged proteins and replaces them with newly synthesized, functional proteins [1,2]. Protein turnover is important in wound healing [3] and aging [4,5]. Measuring protein turnover is essential for understanding cellular renewal, adaptation, and disease response [6,7,8]. Metabolic labeling with deuterium-enriched drinking water combined with liquid-chromatography coupled with high-resolution, high-accuracy mass spectrometry (LC-MS) is a high-throughput approach to determine the turnover rates of individual proteins in vivo [9,10]. Deuterated water labeling is one of the few labeling approaches (such as ^15^N labeling [11,12,13] or labeling with heavy essential amino acids [14,15,16], e.g., [^13^C_6_]Lysine) used for the stable isotope labeling of samples to study with LC-MS [17,18,19]. Some of the features of deuterated water labeling are its ease of use, which includes no need for dietary adaptation, safety (in the low enrichment of deuterium), and cost-effectiveness [10,20].

**Figure 1:**
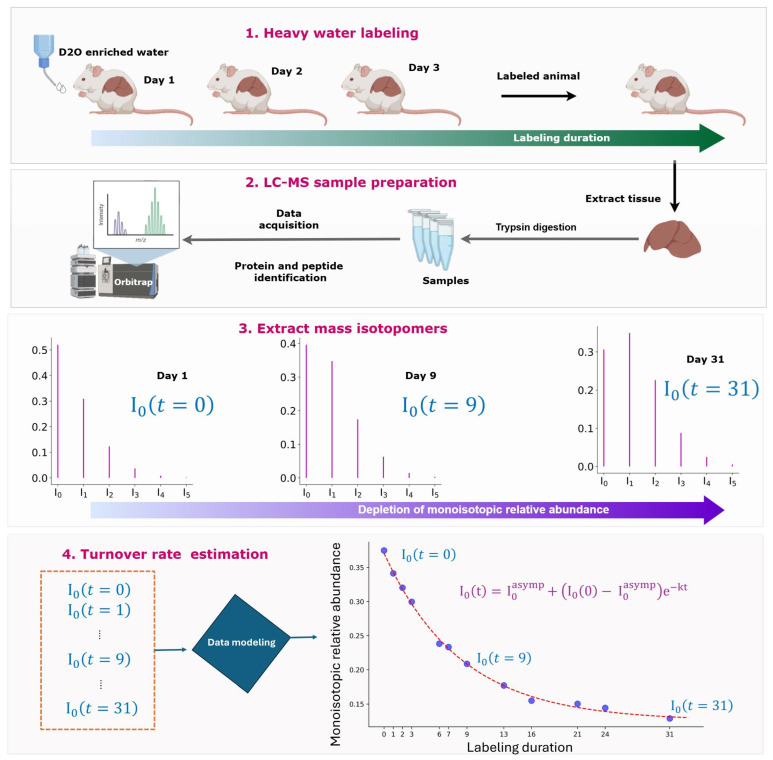
Experimental and data processing workflow for studying protein turnover using metabolic labeling with deuterated water followed by liquid-chromatography coupled with mass spectrometry analysis of the labeled samples. Living organisms are labeled with deuterium-enriched (up to 8% *v*/*v*) drinking water. Tissues are collected at predetermined labeling durations, and proteins are extracted and digested into peptides using trypsin. Mass spectrometric analysis provides peptide/protein identifications. Software tools are used to extract the isotope profiles of peptides and determine the turnover rates from the time series data of the monoisotopic relative abundance.

The deuterium in deuterated drinking water mostly labels the side chains of non-essential amino acids [21,22]. The number of labeling sites is often referred to as the number of exchangeable hydrogens (N_EH_) of an amino acid. The deuterium incorporation of a peptide/protein at any given labeling duration depends on the N_EH_ values (mostly obtained as the sum of the N_EH_ values of amino acids present in a peptide), the turnover rate of the protein, and the body water enrichment in the deuterium. The enrichment is mainly quantified from the isotope distribution of a peptide [23]. The time course of the depletion of the relative abundance (RA) of the monoisotope is used to determine the turnover rate. Thousands of proteins are quantified using the workflow, and software tools are developed to automate the turnover rate computations in high-throughput experiments [24,25,26], Figure 1.

The turnover rates of the peptides of a protein exhibit variability. It has been argued that this variability exceeds the levels that can be attributed to technical fluctuations [27]. This variability is an important factor in determining the significance of changes in the turnover rates of a protein obtained from samples under different conditions. Systematic variations caused by non-biological factors compound estimations of the significance of changes. In studies of proteome turnover using deuterated water-labeled samples, it has been observed that the turnover rates of peptides depend on the N_EH_ value of the peptide. Here, we explore and quantify the dependence of the turnover rate on the N_EH_ using the traditional approach. We propose an alternative approach for turnover rate computation, which removes the dependence on N_EH_ and reduces the variances in the turnover rates of the peptides of a protein. Our approach is computational. It uses the form of the time-course equation for turnover rate estimation to propose a change that makes the solution to the equation independent of N_EH_.

## 2. Results and Discussion

For the label enrichment analysis of the deuterium-labeled peptides, the number of exchangeable hydrogens (the number of labeling sites) plays an important role [28]. It is one of the determinants of label enrichment, along with the turnover rate, labeling duration, and body water enrichment in deuterium. However, the turnover rate of a peptide should be a characteristic of the protein only and be independent of the N_EH_ value. In other words, different peptide sequences originating from the same protein should have similar turnover rates (within technical variations), regardless of the differences in their N_EH_ values. However, the analyses [29,30] of turnover rates using deuterated water labeling showed that they correlate with the N_EH_ numbers. This correlation contributes to the variation in the turnover rates of the peptides of a protein. Understanding and removing the sources of systematic variations that are not due to the biological/environmental factors will improve the statistical significance of the computed turnover rates. Below, we analyze the dependence of the turnover rates of peptides on the N_EH_ values and propose an approach to remove the dependence.

**Figure 2:**
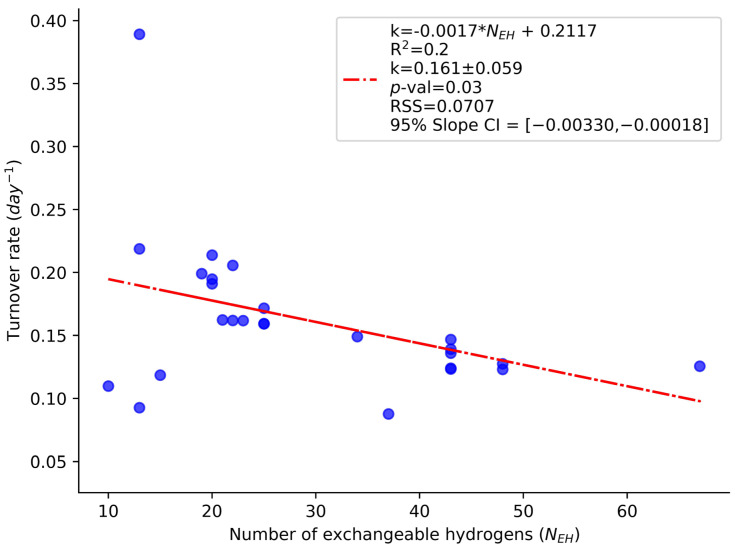
Relationship between the peptide turnover rate and the number of exchangeable hydrogens (N_EH_) for peptides of the murine CH60 protein. The scatter plot shows a consistent dependence of peptide turnover rates on N_EH_. The negative slope of the red regression line indicates a detectable relationship. This trend contradicts the biological expectation that turnover rate should be independent of N_EH_, revealing a technical or computational artifact in the estimation process.

The N_EH_ dependence of the turnover rates is demonstrated in the example of the turnover rates of the peptides of the murine liver CH60 protein (60 kDa heat shock protein, mitochondrial). This protein is one of the abundant proteins in the liver. Its label enrichment and turnover rate are quantified by 51 peptides. The distribution of the turnover rates of CH60 peptides as a function of their N_EH_ values is shown in Figure 2. As seen from the figure, the turnover rates of the peptides of the same protein are dependent on the N_EH_ values. The slope of the linear regression was statistically significant (*p* = 0.0305). This empirical observation agrees with the analyses following Equation (6) for the proportion of the unlabeled species relative to the plateau of labeling. Thus, the theoretically calculated relative proportion of unlabeled peptides (at the protein half-life) for N_EH_ = 15 was 43%, while the respective value for N_EH_ = 25 was 38%. The observation for the CH60 protein generalizes to the whole liver proteome, which is demonstrated in Figure S1. The turnover rates are the derivative parameters—these are obtained from the modeling of the time course of the monoisotopic RA. The monoisotopic RA of a peptide is expected to depend on the peptides’ N_EH_ value, as can be seen from Equation (3). However, the enrichment in deuterium, p_X_(t), can be viewed as a property of a protein and only dependent on the protein turnover rate, labeling duration, and body water enrichment, p_W_. Therefore, p_X_(t) as computed by Equation (5) could be thought of as independent of N_EH_ and as the same for all peptides of a protein at any given duration of labeling.

**Figure 3:**
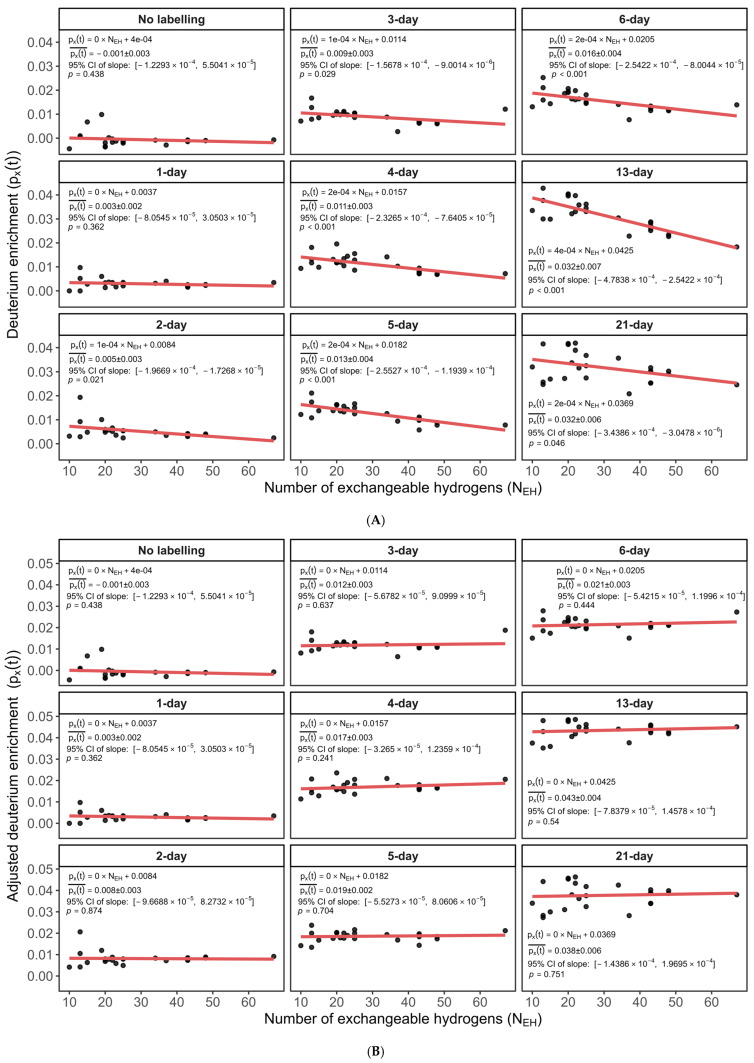
Scatter plots of p_X_(t) (the y-axis) versus N_EH_ (the x-axis) across various labeling time points: (**A**) before adjustment for peptides for the murine CH60 protein. Each subplot corresponds to a distinct labeling duration (time point) and shows a consistent negative dependency. Red lines represent the linear regression of p_X_(t) on N_EH_. This persistent trend implies that p_X_(t) values are systematically biased by N_EH_ and can propagate systematic error into turnover rate calculations. (**B**) After adjustment for peptides of the murine CH60 protein. Each subplot depicts p_X_(t) values after post hoc adjustment, showing a flat (zero-slope) relationship with N_EH_. Red lines indicate linear regression fit, demonstrating successful removal of N_EH_-dependent bias.

As seen in Figure 2, the turnover rates of the peptides of the murine CH60 protein exhibit a negative trend related to the N_EH_ values, indicated by the negative slope from the linear regression analysis. This experimental dependence contradicts the expected biological assumption that protein turnover rates should solely reflect inherent protein characteristics and be independent of peptide-specific attributes such as N_EH_. We further analyzed the relationship between the inferred deuterium enrichment (p_X_(t)) and N_EH_ across various labeling durations (time points) in peptides from the murine CH60 protein. As seen in Figure 3A, there is a consistent dependency between p_X_(t) and N_EH_. The figure shows the deuterium enrichment (the y-axis) as a function of the N_EH_ values (the x-axis) of 21 CH60 peptides for nine different labeling durations. The summary statistics of the regressions are shown as in-figure explanations. The slope of the regression of the peptide p_X_(t) on the peptide N_EH_ values is non-zero for all labeling durations longer than one day. For these labeling durations (>1 day), the 95% confidence intervals (CI) of the slopes did not contain zero, and all were statistically significant with *p*-values less than 0.05.

The systematic bias observed in Figure 3A compounds with other sources of error, such as cumulative technical inaccuracies, including instrumental noise, truncation errors, or minor inaccuracies in isotope peak quantification. Because these errors accumulate, they introduce measurable biases into the subsequently computed enrichment values.

Rather than modifying the raw experimental data directly, we opted to apply a computational correction approach. Specifically, we performed a regression analysis of p_X_(t) against N_EH_ and subtracted the N_EH_-dependent component from the original p_X_(t) values. Figure 3B visually confirms that this adjustment successfully eliminates the dependence of p_X_(t) on N_EH_ in peptides from the murine CH60 protein, as peptides align consistently along a baseline with a zero slope. As seen from the summary statistics in the figure, the 95% CI of the slope (on N_EH_) of p_X_(t) for all labeling durations includes zero, and the corresponding *p*-values are 0.2 or higher. Therefore, the null hypothesis (p_X_(t) is independent of N_EH_) cannot be rejected. The p_X_(t) values at the long labeling durations correspond to the body water enrichment levels, as can be seen from the results for 15 and 21 days of labeling. This shows that the original meaning of p_X_(t) as the excess labeling in deuterium is still relevant in the new approach. The R^2^ values for the adjusted p_X_(t) are not shown (Figure 3B) as R^2^ is not a relevant indicator for a theoretical fit, when the slope is zero [24]. It is seen from the R^2^ of the fit to the data from the unlabeled sample (0-day labeling), as shown in Figure 3A.

**Figure 4:**
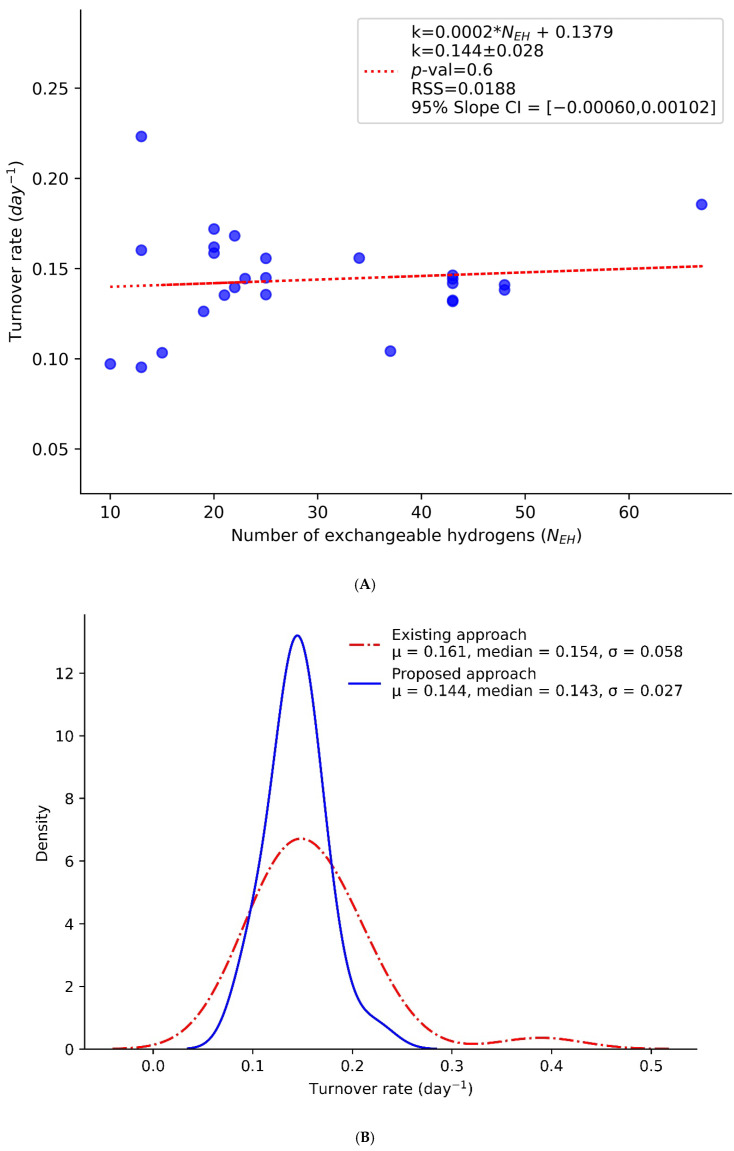
Turnover rates of CH60 peptides after p_X_(t) adjustment show no observable dependence on N_EH_. (**A**) Scatter plot of turnover rates (y-axis) versus N_EH_ (x-axis) demonstrates a zero-slope regression line, indicating the effectiveness of the computational adjustment (correction) in eliminating or reducing N_EH_-associated bias. (**B**) The comparison of distributions of turnover rates before (red dashed line) and after (blue solid line) p_X_(t) adjustment shows more than a 50% reduction in standard deviation, indicating the enhanced precision of the turnover estimates.

The adjustment of p_X_(t) propagates forward to the estimation of turnover rates, as shown in Figure 4A. The figure shows the turnover rates (y-axis) of peptides as a function of their N_EH_ values (x-axis). The adjusted turnover rates do not depend on N_EH_ values in the peptides of the murine CH60 protein, unlike the dependency observed in Figure 2. This approach has significantly reduced the variability in turnover rates among the constituent peptides of the protein. As shown in Figure 4B, the standard deviation of the peptides’ turnover rates decreased by more than 50%, dropping from 0.058 to 0.027 after implementing the proposed method. This improvement will enhance the confidence intervals and statistical significance of the estimated turnover rates for the protein. We note that even though the modeling presented in this work is based on a mathematics rather than a physics rationale, the interpretations of the resulting turnover rates (from Equation (7)) and excess enrichment in deuterium are the same as in the original approach. Thus, despite the transformations used in the kinetic equation, Equation (7), the resulting p_X_(t) values are representative of the body water enrichment, as seen in Figure 3B.

**Figure 5:**
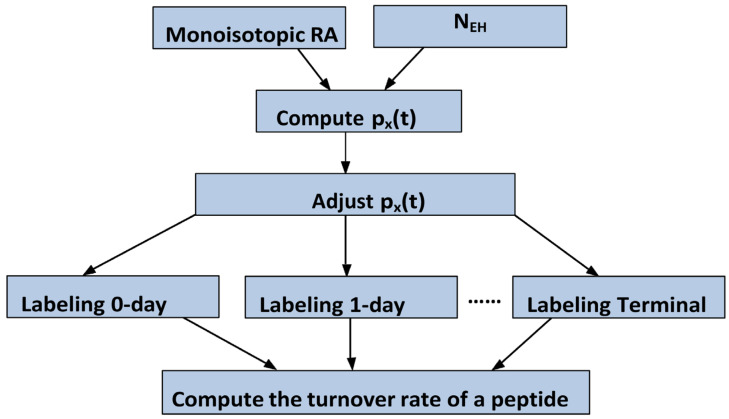
The flowchart of the data processing workflow was used in this work to remove the dependence of the turnover rates on the number of exchangeable hydrogens (N_EH_). At every labeling duration, the monoisotopic relative abundance of a peptide and its N_EH_ are used to compute the excess enrichment in deuterium (p_X_(t)). The linear regression of p_X_(t) on N_EH_ is used to determine the linear component. The linear dependence is removed in the adjusted p_X_(t), while preserving the original linear model error. The time course of the ratio of adjusted p_X_(t) to N_EH_ is fitted to an exponential function to determine the turnover rate.

The flowchart of our approach is shown in Figure 5. It has two main steps for each peptide: (1) removal of the dependence of the excess enrichment in deuterium, p_X_(t), on N_EH_ at every labeling time; (2) modeling peptide/protein turnover rates using the time course of the ratio (p_X_(t)/N_EH_) of the excess enrichment in deuterium to N_EH_, Equation (7).

To further validate our findings comprehensively, we conducted a similar analysis across the entire liver proteome. We calculated and compared p_X_(t) values before and after adjustment, along with their corresponding turnover rates. The detailed results from these extensive analyses are presented in Supplementary Figures S1A,B and S2A,B. Figure S1A and S1B show scatter plots of p_X_(t) values against the N_EH_ for peptides of the liver proteome across multiple labeling time points before and after computational adjustment, respectively. Each subplot in Figure S1A illustrates a clear and consistent dependency of p_X_(t) on N_EH_, similar to the artifacts observed for CH60 protein peptides (Figure 3A). This indicates a systemic bias resulting from cumulative technical inaccuracies. In contrast, Figure S1B displays a flat distribution of p_X_(t) against N_EH_ at all labeling durations, with regression lines exhibiting zero slopes. This confirms that the N_EH_-dependent bias was successfully removed from the deuterium enrichment values. The observed patterns closely resemble those demonstrated in Figure 3B for the CH60 protein, validating the generalizability of the computational adjustment method across the broader liver dataset.

**Figure 6:**
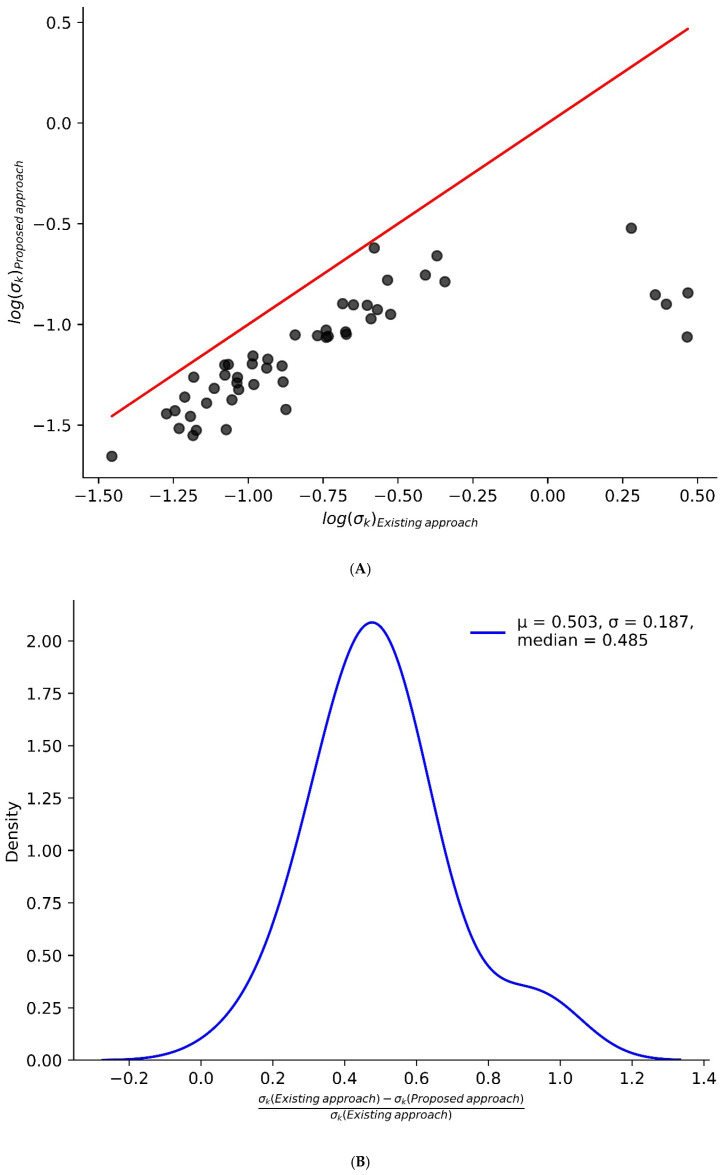
The proposed computational approach significantly reduces intra-protein variability. (**A**) Scatter plot comparing the standard deviation of protein turnover rates estimated using the existing method (x-axis) and the proposed method (y-axis). Most points fall below the unity line, indicating a reduction in variability with the proposed approach. (**B**) A density plot shows the relative difference in standard deviation between the two methods, further demonstrating the overall decrease in intra-protein variability.

Table S1 summarizes the mean standard deviations of peptide turnover rates before and after computational adjustment for 40 liver proteins. The results clearly demonstrate a substantial reduction in the variability of turnover rate estimates within peptides of the same protein due to our proposed approach. For instance, the standard deviation for CH60 decreased from 0.0588 to 0.0276, and for FAS, from 0.2696 to 0.1176. Figure 6A presents a scatter plot comparing the standard deviation of turnover rates calculated using the existing approach (x-axis) and the proposed approach (y-axis). The plot clearly shows that the new approach significantly reduces the standard deviation of turnover rates for several proteins. This reduction is further illustrated in Figure 6B, which features a density plot of the relative difference between the standard deviations obtained from the two methods ($\frac{\sigma_{k}\;\left(Exisiting\;approach\right)-\sigma_{k}\;(Proposed\;approach)\;}{\sigma_{k}\;\left(Exisiting\;approach\right)}$). The mean and standard deviation of the relative difference are approximately 0.5 and 0.18, respectively. Overall, the proposed method reduces the standard deviation of turnover rates by around 50%. This reduction in intra-protein turnover rate variability demonstrates that the proposed approach effectively reduces biases related to N_EH_, thereby significantly enhancing statistical power and biological interpretability in peptide-level turnover rate estimations. The R scripts used for the analysis of the proposed method are available on GitHub (v.1.0.0): https://github.com/henockmamo54/Turnover_Rate_NEH_dependence (accessed on 24 June 2025).

The above analysis demonstrated that our computational approach to modeling protein turnover based on Equation (7) effectively eliminated (reduced) the bias associated with N_EH_ while preserving the integrity and biological validity of the dataset. Thus, this method may help to enhance the accuracy and biological validity of turnover rate estimations. For example, we compared the turnover rates of proteins from murine liver and kidney tissues [20]. Both are fast protein turnover tissues. Identifying proteins which show statistically significant changes in turnover between these tissues requires that the precisions of turnover rate estimations be high. For example, murine SDHB (succinate dehydrogenase iron-sulfur subunit) shows fast turnover rates 0.103 day^−1^ and 0.126 day^−1^ in kidney and liver tissues, respectively. However, if the original approach to determine protein turnover rates is used, the *p*-value from the t-test comparing the turnover rates of peptides is equal to 0.19 and is not significant. The approach proposed in this work results in turnover rates of 0.101 day^−1^ (kidney) and 0.123 day^−1^ (liver). The t-test becomes statistically significant (*p*-value = 0.02) The significance is achieved mainly due to the reduction in the standard deviations of protein turnover in the kidney (from 0.028 in the original method to 0.027 in the proposed approach) and liver (from 0.021 to 0.017). The corresponding box plots are shown in Supplementary Figure S3A and Figure 3B.

In general, the proposed approach may be useful in contexts where subtle changes in protein turnover rates are expected to result from different biological conditions. For example, in heart failure [31], biologically significant but moderate changes may be obscured. Our proposed approach may enhance the detection of these subtle changes, thereby improving biological interpretability.

Some of the limitations in our approach are noted. Thus, if Equation (6) is a fractional synthesis, Equation (8) does not represent a specific physical property. It is deducted simply based on a numerical analysis for removing the linear part of the dependence of the turnover rate on N_EH_. In addition, while the systematic effects of N_EH_ were removed, the general experimental error (due to sample processing, mass spectral data acquisition, co-elutions, etc.) on the turnover rate remained, as seen in Figure 4.

The adjusted formula, (Equation (7)), is empirically derived, rather than representing a mechanistic biological model. However, as shown in the Materials and Methods Section, by comparing Taylor’s expansions of Equations (4) and (7), we concluded that the difference between the expansions results from the relationship between the N_EH_ value and p_W_. Thus, the equations are equivalent if N_EH_* ×p_W_ ≤ 1. This requirement is necessary for linear expansion in Equation (4). However, this condition is not satisfied for rodent enrichment levels (0.03–0.05) and peptides with N_EH_ > 20. Equation (7) places a milder restriction on N_EH_ for linear expansion: N_EH_ ×p_W_^2^ ≤ 1. This requirement is satisfied for N_EH_ values up to 400 which makes it applicable to practically all peptides.

## 3. Materials and Methods

### 3.1. Turnover Rates Are Determined from the Time Course of Monoisotopic RA

Living organisms are labeled with deuterium using deuterated (enriched up to 8% *v*/*v*) drinking water [32], as shown in Figure 1. At the predefined labeling durations, tissues are collected, and protein samples are prepared. The proteins are digested into peptides using proteases, e.g., trypsin. Peptide samples are analyzed in LC-MS, and their tandem mass spectra are used to determine the peptide sequences from protein sequence databases [33]. Peptide turnover rates are determined from the LC-MS time-course (in the labeling duration domain) data of deuterated samples. As the newly synthesized non-essential amino acids are incorporated into proteins, the isotope distributions of the peptides resulting from the trypsin digestion of the proteins shift to heavy masses. The depletion of the monoisotopic RA is modeled as an exponential decay function shown below [34]:

(1) $$I_{0}\left(t\right)=I_{0}^{asymp}+\left({I_{0}\left(0\right)-I}_{0}^{asymp}\right)e^{-kt}$$

where t is the labeling duration, I_0_(0) is the monoisotopic RA of natural peptide (with the natural deuterium abundance), I_0_^asymp^ is the monoisotopic RA achieved at the plateau of labeling, and k is the turnover rate. Since the labeling with deuterium is not completed (p_W_ is typically around 2–4%), the asymptotic labeling in deuterium does not fully deplete the monoisotopic RA of a peptide. Instead, I_0_^asymp^ is non-zero and is determined from the body water enrichment level, p_W_, the number of exchangeable hydrogens (the labeling sites), N_EH_, and the natural monoisotopic abundance, I_0_(0):

(2) $$I_{0}^{asymp}=I_{0}(0){\left(1-\frac{p_{W}}{1-p_{H}}\right)}^{N_{EH}}$$

In the above formula, pH is the natural abundance of 2H. To determine the turnover rate, k, non-linear least-squares regression is used to fit the time course of the experimental data for the monoisotopic RA to the theoretical distribution in Equation (1). The turnover rate, as determined from Equation (1), exhibits dependence on the N_EH_ number. This can be seen from the viewpoint of the averaged enrichment in deuterium of a peptide at the labeling duration t, p_X_(t). Assuming the enrichment, p_X_(t), it can be shown [32] that the monoisotopic RA at labeling duration t is

(3) $$I_{0}(t)=I_{0}(0){\left(1-\frac{p_{X}(t)}{1-p_{H}}\right)}^{N_{EH}}$$

Using Equation (3), one can model the turnover rate estimation as a non-linear fitting of the excess enrichment in deuterium, p_X_(t), to a growth function (for p_X_(t)):

(4) $${\left(1-\frac{p_{X}(t)}{1-p_{H}}\right)}^{N_{EH}}={\left(1-\frac{p_{W}}{1-p_{H}}\right)}^{N_{EH}}+\left(1-{\left(1-\frac{p_{W}}{1-p_{H}}\right)}^{N_{EH}}\right)e^{-kt}$$

The experimental p_X_(t) values are obtained from the monoisotopic RA:

(5) $$p_{X}\left(t\right)=(1-p_{H})\left(1-{\left(I_{0}(t)/I_{0}(0)\right)}^{{1/N}_{EH}}\right)$$

Equations (1) and (4) produce identical results for the turnover rate, k, given the same time course of I_0_(t). Equation (3) allows exploration of the dependence of the turnover rate on the N_EH_ values. For example, at the labeling duration corresponding to the protein half-life, half of the proteins are newly synthesized, and the average p_X_(t) equals to p_W_/2. The fractional synthesis, f(t), the proportion of newly synthesized proteins, is expected to be halved at the protein half-life:

$$f(t_{1/2})=\frac{{I_{0}\left(t_{1/2}\right)-I}_{0}^{asymp}}{{I_{0}\left(0\right)-I}_{0}^{asymp}}=1-e^{-kt_{1/2}}=1/2$$

Using Equation (3) for I_0_(t_1/2_), we obtain

(6) $$\frac{{\left(1-\frac{p_{W}}{2(1-p_{H})}\right)}^{N_{EH}}-{\left(1-\frac{p_{W}}{1-p_{H}}\right)}^{N_{EH}}}{1-{\left(1-\frac{p_{W}}{1-p_{H}}\right)}^{N_{EH}}}=1/2$$

If the model of the turnover rate was independent of the N_EH_, the right and left sides of Equation (6) should be equal for every pair of N_EH_ and p_W_. However, a simple computation shows that the results from the left-hand side of Equation (6), when used in combination with practical body water enrichment ranges (p_W_ ≈ 0.02–0.04), depend on N_EH_. Specifically, if one assumes p_W_ = 0.04, the ratio is equal to 0.43 for N_EH_ = 15 and 0.38 for N_EH_ = 25, respectively. It is noted that for smaller body water enrichment levels, p_W_ ≤ 0.01, Equation (6) becomes more accurate. For example, for p_W_ = 0.01, the ratio on the left-hand side of Equation (4) is 0.48 and 0.47 for N_EH_ = 15 and N_EH_ = 25, respectively.

The N_EH_ dependence of the turnover rate (and fractional synthesis as well) results from the formula for the time dependence of the monoisotopic RA, Equation (3). When the formula is expanded into the power series, the leading term is linearly dependent on N_EH_. With this observation, we propose an alternative approach to model the data and obtain turnover rates that are less dependent (practically independent) on N_EH_. We propose to replace p_X_(t) in Equation (4) with p_X_(t)/N_EH_. The functional form of the model then becomes

(7) $${\left(1-\frac{p_{X}(t)}{N_{EH}\left(1-p_{H}\right)}\right)}^{N_{EH}}={\left(1-\frac{p_{W}}{N_{EH}\left(1-p_{H}\right)}\right)}^{N_{EH}}+\left(1-{\left(1-\frac{p_{W}}{N_{EH}\left(1-p_{H}\right)}\right)}^{N_{EH}}\right)e^{-kt}$$

At the half-life (kt = ½), Equation (7) is practically independent of N_EH_, which implies that

(8) $$\frac{{\left(1-\frac{p_{W}}{2N_{EH}(1-p_{H})}\right)}^{N_{EH}}-{\left(1-\frac{p_{W}}{N_{EH}(1-p_{H})}\right)}^{N_{EH}}}{1-{\left(1-\frac{p_{W}}{N_{EH}(1-p_{H})}\right)}^{N_{EH}}}≅1/2$$

For example, the left-hand side of Equation (8) equals 0.4953 and 0.4952 for N_EH_ values of 15 and 25, respectively (p_W_ = 0.04). In general, the kinetics from Equation (7) results in similar ratios of newly synthesized to old peptides (independent of the peptides’ N_EH_ values) at the labeling duration corresponding to the protein half-life. This result from Equation (8) shows that unlike Equation (4), the solution for p_X_(t) obtained from Equation (7) theoretically does not imply N_EH_ dependence.

We note that the above result (about the independence of p_X_(t) on N_EH_ values at half-life) is true for all labeling durations. This can be seen by Taylor’s expansion [35] of each term containing N_EH_ power in Equation (7) and keeping only the first power in p_X_(t) and p_W_. Thus, one will obtain for p_X_(t)

$$p_{X}(t)=p_{W}\left(1-e^{-kt}\right)$$

The above expression does not contain N_EH_. It demonstrates, for small values of p_W_ (note that p_X_(t) ≤ p_W_, as seen from the above expression), such that $N_{EH}\;*\;p_{w}^{2}\ll 1$, p_X_(t) from Equation (7) is independent of N_EH_. On the other hand, for a similar result, Equation (4) requires a stronger condition on p_W_, specifically that $N_{EH}\;*\;p_{W}\ll 1$. As was mentioned, p_W_ in the labeling of rodents reaches 0.04–0.05. The N_EH_ values of peptides can be up to 60, as shown in Figure 2 and Figure 3 (the x-axis). Therefore, the condition $N_{EH}\;*\;p_{W}\ll 1$ will not be satisfied for large values of N_EH_. On the other hand, $N_{EH}\;*\;p_{w}^{2}\ll 1$ is satisfied with large and small values of N_EH_.

### 3.2. Mass Spectral Datasets

For tests with datasets obtained by liquid-chromatography coupled with mass spectrometry (LC-MS), we used murine liver datasets [32]. We selected the liver dataset due to its central role in metabolism and its rapid protein turnover dynamics, making it a well-characterized model for proteomic studies involving deuterium labeling and turnover analysis. In addition, liver proteome datasets exhibit broad peptide diversity, high dynamic range, and extensive protein representation, which makes them ideal for robust modeling and statistical validation. The experiments and data accessibility in the public repository are described in the original publication. Briefly, experimental datasets were acquired in data-dependent acquisition mode with high mass resolution and accuracy in MS1 (recorded in Orbitrap). The tandem mass spectra were recorded using an ion trap mass analyzer (Orbitrap Eclipse, Thermo Fisher Scientific, Waltham, MA, USA). Raw mass spectral data were converted to mzML format [36] using the MSConvert tool [37] of proteowizard [38] version 3.0.22048. Peptides were identified from tandem mass spectra using the database search engine Mascot [39] (Matrix Science, Boston, MA, USA) version 2.5. SwissProt (v.2.7.0) [40] (12 February 2022 release) was used as a protein sequence database. The database search parameters were set as Mus Musculus for taxonomy, 15 ppm (parts per million) for mass tolerance of precursors, 0.6 Da as a mass tolerance of fragment ions, fixed modification of Cys residues with carbamidomethylation, variable oxidation of Met (+15.9949), and variable protein N-terminal acetylation. # ^13^C was set to 2, which allows for 0/1/2 isotope errors. This setting permits the selection of higher mass isotopomers, which is important for deuterated water samples. Trypsin was specified as the protease with up to two missed cleavage sites. Only the first-ranked peptides (Mascot bold red peptides) matching the tandem mass spectra were retained. The false discovery rate (FDR) of identifications was controlled by using reversed sequences [41]. The database search results were exported in the mzIdentML file format [42] and were used as inputs to d2ome.

## 4. Conclusions

In this study, we demonstrated that conventional computations of peptide turnover rates from deuterium-labeled datasets exhibit a systematic dependence on the number of exchangeable hydrogens (N_EH_). This artifact inflates the variability in turnover rate estimates across peptides derived from the same protein and reduces the biological interpretability of the results. We provide both theoretical and empirical evidence showing that this dependence derives from the way the turnover rate is mathematically formulated and is further amplified by technical errors.

To address this issue, we propose an alternative two-step approach. First, we introduce a mathematical model for turnover rate estimation that removes the dependence on N_EH_ values. Second, we apply a post hoc computational adjustment of the excess enrichment in deuterium (p_X_(t)) against N_EH_ and remove the N_EH_-dependent component without modifying the raw experimental data. This adjustment corrects the artificial N_EH_ dependency introduced by the modeling and amplified by technical noise or quantification imprecision. Finally, when the correction is propagated in the turnover rate computation, it eliminates the N_EH_ dependence of the resulting turnover rate estimates. This approach results in biologically accurate and statistically unbiased results.

We validated this approach using a murine liver dataset and showed it removed N_EH_-related biases in deuterium enrichment values (p_X_(t)) and the derived turnover rate estimates. Furthermore, we performed a comprehensive analysis across the full liver datasets that confirms that our approach consistently improves the reliability of turnover measurements across peptides.

## Data Availability

The original data presented in the study is publicly available in the MassIVE data repository [43] at http://massive.ucsd.edu (accessed on 15 January 2025) with the identifier MSV000090148.

## Supplementary Materials

The following supporting information can be downloaded at: https://www.mdpi.com/article/10.3390/ijms26136398/s1.
