# Supplementary material for: Turnover Rates and Numbers of Exchangeable Hydrogens in Deuterated Water Labeled Samples
Source: Int J Mol Sci. 2025 Jul 3;26(13):6398. doi: 10.3390/ijms26136398 (PMC12249490; doi:10.3390/ijms26136398)
Supplement: Supplementary file 1 [file ijms-26-06398-s001.zip › ijms-3676265-supplementary.pdf]

Supporting Information for

Turnover Rates and Numbers of Exchangeable Hydrogens in Deuterated Water Labeled Samples

Henock M. Deberneh[1,#], Ali Bagherinia[1,#], and Rovshan G. Sadygov[1,*]

[1]Department of Biochemistry and Molecular Biology

The University of Texas Medical Branch

301 University of Blvd

Galveston, TX 77555

Abbreviations: LC–liquid chromatography; MS–mass spectrometry; SD–standard deviation.

Keywords: protein turnover, deuterated water labeling, the number of exchangeable hydrogens.

[#]These authors contributed equally to this work.

*To whom the correspondence should be addressed, email: rgsadygo@utmb.edu, telephone: (409)772-3287

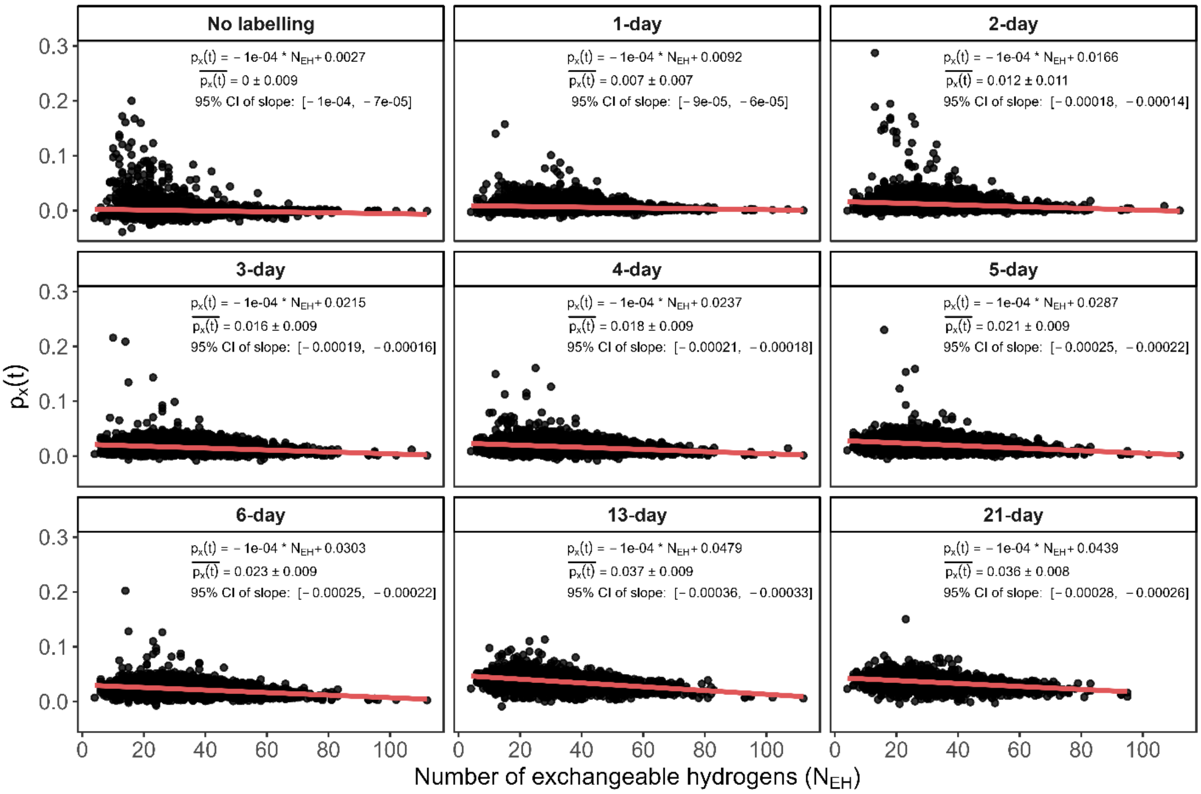

No labelling
1-day
2-day
3-day
4-day
5-day
6-day
13-day
21-day
px(t) = − 1e-04 * NEH + 0.0027
px(t) = 0 ± 0.009
95% CI of slope: [− 1e-04, − 7e-05]
px(t) = − 1e-04 * NEH + 0.0092
px(t) = 0.007 ± 0.007
95% CI of slope: [− 9e-05, − 6e-05]
px(t) = − 1e-04 * NEH + 0.0166
px(t) = 0.012 ± 0.011
95% CI of slope: [− 0.00018, − 0.00014]
px(t) = − 1e-04 * NEH + 0.0215
px(t) = 0.016 ± 0.009
95% CI of slope: [− 0.00019, − 0.00016]
px(t) = − 1e-04 * NEH + 0.0237
px(t) = 0.018 ± 0.009
95% CI of slope: [− 0.00021, − 0.00018]
px(t) = − 1e-04 * NEH + 0.0287
px(t) = 0.021 ± 0.009
95% CI of slope: [− 0.00025, − 0.00022]
px(t) = − 1e-04 * NEH + 0.0303
px(t) = 0.023 ± 0.009
95% CI of slope: [− 0.00025, − 0.00022]
px(t) = − 1e-04 * NEH + 0.0479
px(t) = 0.037 ± 0.009
95% CI of slope: [− 0.00036, − 0.00033]
px(t) = − 1e-04 * NEH + 0.0439
px(t) = 0.036 ± 0.008
95% CI of slope: [− 0.00028, − 0.00026]
px(t)
Number of exchangeable hydrogens (NEH)

Figure S 1A

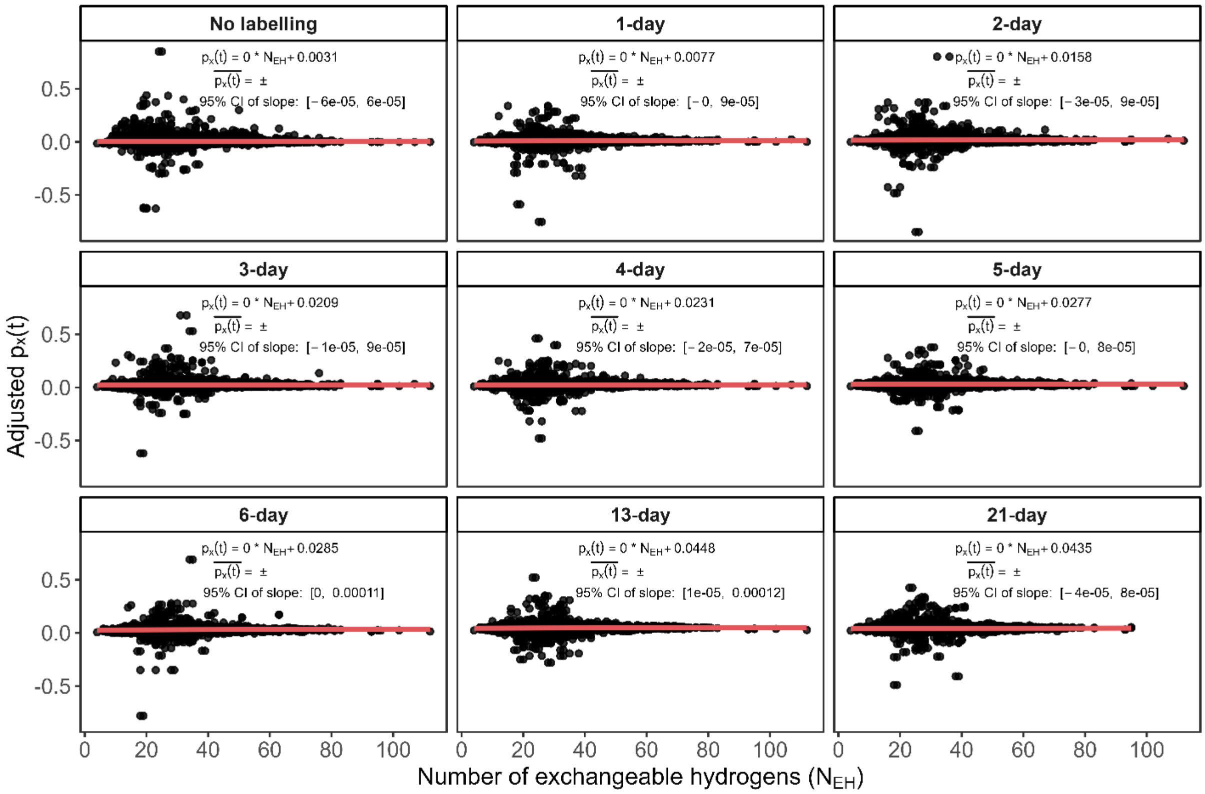

Figure S 1B

Figure S1. Scatter plots of $p_X(t)$ against the number of exchangeable hydrogens (N_{EH}): **(A)** before computational adjustment for peptides from all liver proteins. Each subplot corresponds to a specific labeling duration (time point). The red lines represent linear regression fits, revealing a consistent negative dependency of $p_X(t)$ on N_{EH} across time points. **(B)** After computational adjustment for peptides from all liver proteins. Each subplot corresponds to a specific time point (duration). At each time point, the fitted red regression line (red line) is essentially horizontal (annotated slope = 0). This demonstrates that the post hoc adjustment has effectively eliminated the systematic N_{EH}-dependency observed in Figure S 1A.

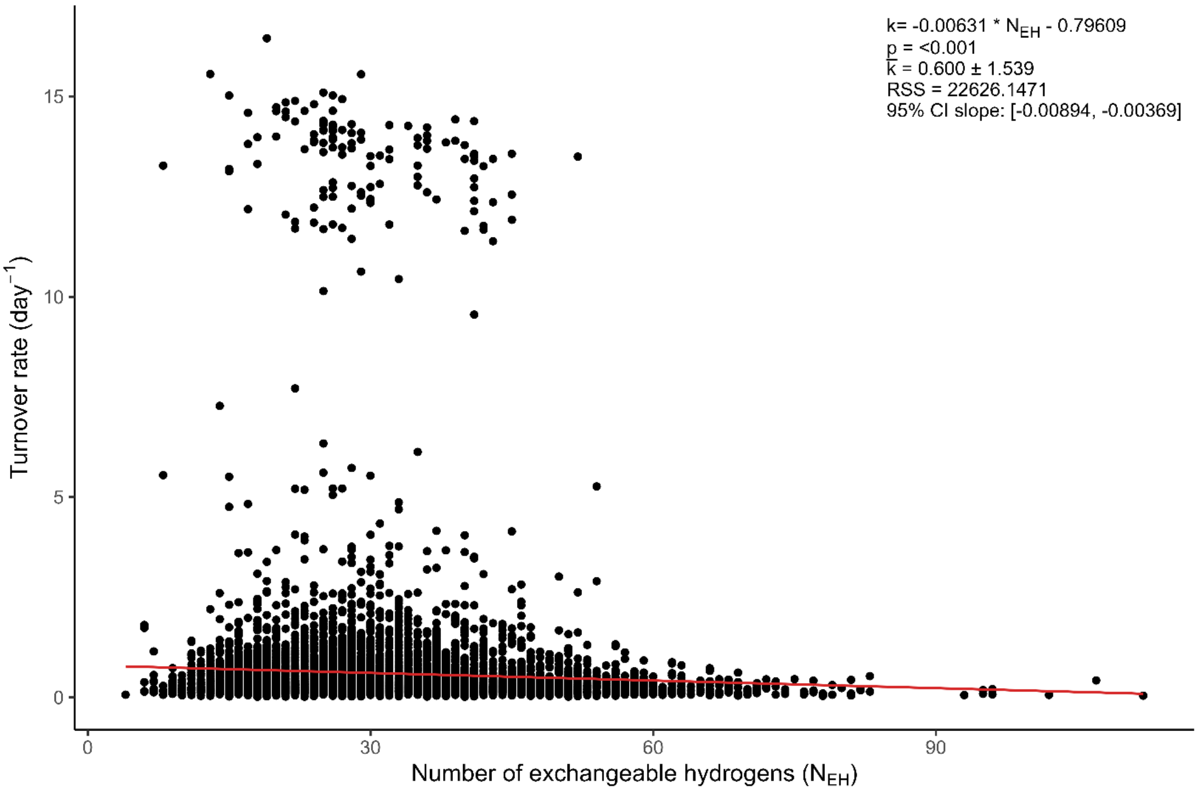
k= -0.00631 * N_EH - 0.79609
p = <0.001
k̄ = 0.600 ± 1.539
RSS = 22626.1471
95% CI slope: [-0.00894, -0.00369]
Turnover rate (day^-1)
Number of exchangeable hydrogens (N_EH)
0
5
10
15
0
30
60
90

Figure S 2A

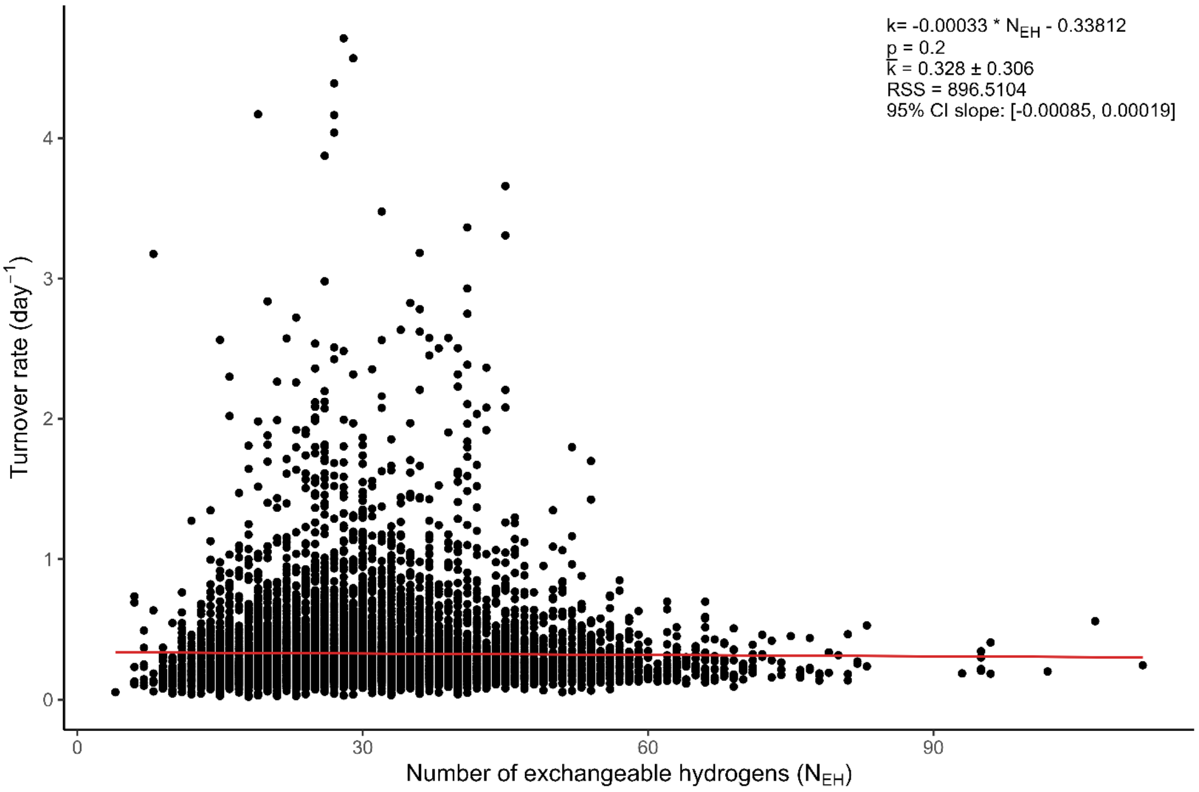

Figure S 2B

Figure S2. Scatter plots of turnover rate against N_{EH}: **(A)** before computational adjustment for peptides from all liver proteins. The red regression line reveals a noticeable dependence of turnover rate on N_{EH}, pointing to (revealing/implying) a systematic bias, **(B)** After computational adjustment for peptides from all liver proteins. This plot shows turnover rates after applying N_{EH} -independent $p_X(t)$ values (as in Supplementary Figure S1-B). The near-zero slope of the red regression line indicates that the computational adjustment (correction) markedly reduces systematic N_{EH}-related bias, resulting in turnover rate estimates that more accurately reflect intrinsic protein dynamics across the liver proteome.

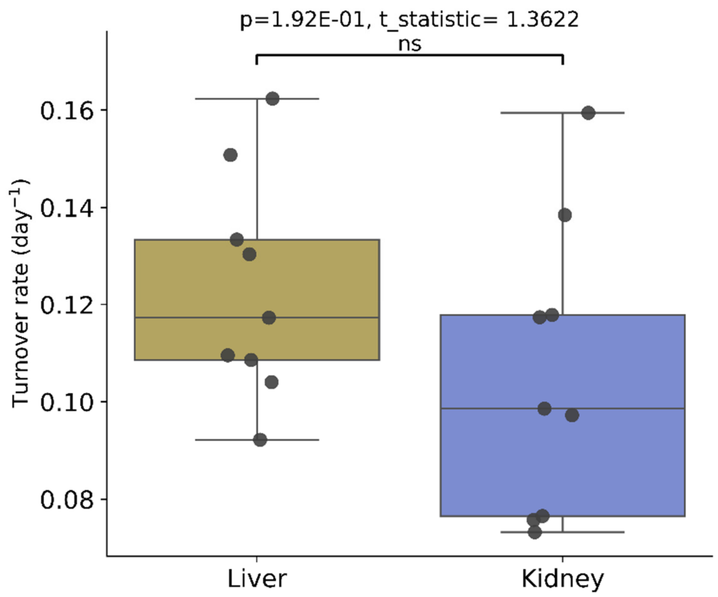

Figure S 3A

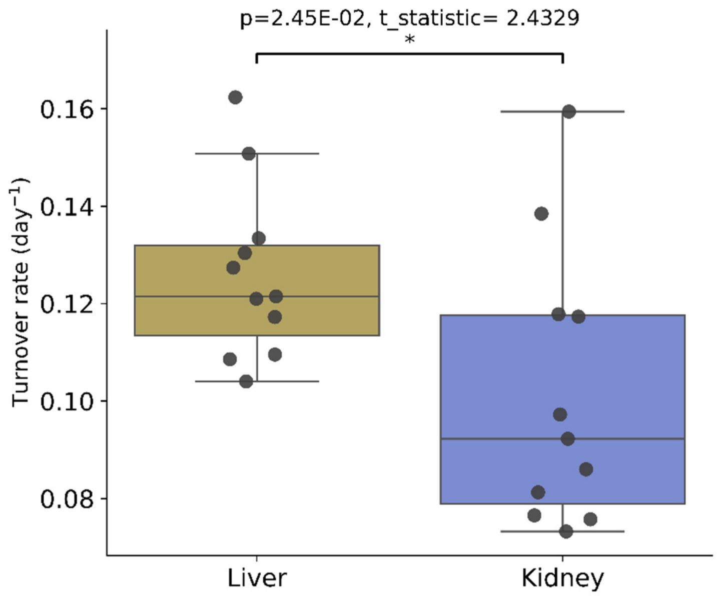

Figure S 3B

Figure S3. Distribution of peptides turnover rates for the SDBH_MOUSE protein in murine liver and kidney tissues **(A)** before and **(B)** after adjustment for $p_x(t)$.

Table S1. The mean standard deviations of peptide turnover rates proposed approach and the traditional method across 40 liver proteins from the liver dataset. The table summarizes the variability in peptide turnover rate estimates for a representative set of liver proteins, comparing the standard deviation (SD) of turnover rates by the proposed approach and the traditional method. Each row corresponds to a protein, reporting the number of peptides used and the respective SD values. The consistent reduction in the proposed approach SD across proteins demonstrates that the two-pass proposed approach reduces non-biological variability caused by NEH dependence and enhances the accuracy and statistical reliability of turnover estimations across peptides of the same protein.

Protein Name	Number of Peptides	Turnover rate (traditional method)	Turnover rate (proposed method)	SD of turnover rate (traditional method	SD of turnover rate (proposed method)
FAS_MOUSE	63	0.610629524	0.420598182	0.26963866	0.118401868
AL1L1_MOUSE	51	0.354352549	0.280895924	0.085716346	0.063358949
SBP1_MOUSE	45	0.287787556	0.237411966	0.104263372	0.050386443
HYES_MOUSE	43	0.330786977	0.267724517	0.09155091	0.05127775
PYC_MOUSE	42	0.21364619	0.186361822	0.129629498	0.062283677
ALBU_MOUSE	40	0.32080325	0.282776935	0.130663861	0.051881791
BIP_MOUSE	36	0.489556944	0.330794127	0.170292827	0.087976083
MYH9_MOUSE	36	0.408069722	0.323486312	0.143328796	0.088755668
ATPB_MOUSE	35	0.142632	0.144437461	0.035043875	0.022172253
CLH1_MOUSE	35	0.195028857	0.178631534	0.064075392	0.035007192
DHE3_MOUSE	35	0.221436571	0.174666952	0.298852443	0.112270581
ENPL_MOUSE	35	0.47073	0.334267541	0.181830372	0.09381686
THIM_MOUSE	35	0.476684571	0.216101639	1.899688093	0.300742588
AL1A1_MOUSE	33	0.297774545	0.236662192	0.067055219	0.029836904
CATA_MOUSE	33	1.156329697	0.747855422	0.291626517	0.165971482
M2GD_MOUSE	33	0.158360909	0.159225646	0.053308801	0.03604265
PYGL_MOUSE	32	0.186620938	0.167282398	0.056884246	0.037287999
G3P_MOUSE	31	0.250505484	0.212745563	0.115213826	0.060752002
SARDH_MOUSE	31	0.584262258	0.178210859	2.48609593	0.126027197

F16P1_MOUSE	30	0.356777667	0.270289166	0.092933673	0.04751683
MTP_MOUSE	30	0.412461333	0.29576061	0.249583102	0.124627484
PDIA3_MOUSE	30	0.450716333	0.311530068	0.212391116	0.089413705
ACSL1_MOUSE	29	0.367647931	0.278171559	0.076955488	0.048194258
ALDH2_MOUSE	29	0.527921034	0.376193943	0.18205833	0.086348491
ARLY_MOUSE	29	0.354595172	0.254503118	0.184839698	0.087305426
ECHA_MOUSE	29	0.625872759	0.20659884	2.283180655	0.140294325
AMPL_MOUSE	28	0.315243571	0.247546216	0.092086706	0.054541902
DHSO_MOUSE	28	0.26538	0.221700142	0.061338946	0.043622669
SAHH_MOUSE	28	0.3247025	0.249462679	0.083421306	0.062973402
BHMT1_MOUSE	27	0.25081	0.235049519	0.065370478	0.028130699
HS90A_MOUSE	27	0.578421111	0.388711908	0.22441944	0.125190466
ACOX1_MOUSE	26	1.259882308	0.752193423	0.453306485	0.163008826
ACTB_MOUSE	26	0.224540769	0.194150041	0.084403482	0.03003933
ASSY_MOUSE	26	0.275486154	0.232000211	0.0725314	0.040744785
ATPA_MOUSE	26	0.204903462	0.1781184	0.211411525	0.091799832
CH60_MOUSE	26	0.161207308	0.152998914	0.058764835	0.030485325
HSP7C_MOUSE	26	0.589966154	0.390052349	0.116169551	0.067168612
TKFC_MOUSE	26	0.316456538	0.264139072	0.08819743	0.042254573
EF2_MOUSE	25	0.5145072	0.348893008	0.256925257	0.106746891
SCP2_MOUSE	25	1.2590072	0.819291088	0.426574453	0.21928011
TKT_MOUSE	25	0.1593164	0.166825245	0.263338548	0.239907099
ECHP_MOUSE	24	0.379872083	0.297698547	0.083517918	0.056080066
GABT_MOUSE	24	0.81481375	0.228032488	2.934732104	0.143510561
GRP75_MOUSE	24	0.411965417	0.299943338	0.103658724	0.069807225
GSTP1_MOUSE	24	0.21480375	0.196205454	0.065749193	0.054773751
HS90B_MOUSE	24	0.575272083	0.405592111	0.206580541	0.126823096
ACADV_MOUSE	23	0.510750435	0.373737116	0.390004975	0.175863532
SND1_MOUSE	23	0.318061739	0.249573101	0.133509496	0.037871917
THIKA_MOUSE	23	1.268002174	0.481522154	2.91848857	0.086739626
UD11_MOUSE	23	0.285518261	0.24173374	0.103054579	0.06370873
